# A pilot study demonstrating the impact of surgical bowel preparation on intestinal microbiota composition following colon and rectal surgery

**DOI:** 10.1038/s41598-022-14819-1

**Published:** 2022-06-22

**Authors:** Harika Nalluri-Butz, Matthew C. Bobel, Julia Nugent, Sonja Boatman, Ryan Emanuelson, Genevieve Melton-Meaux, Robert D. Madoff, Cyrus Jahansouz, Christopher Staley, Wolfgang B. Gaertner

**Affiliations:** 1 Department of Surgery, University of Minnesota, Minneapolis, MN USA; 2 Division of Colon and Rectal Surgery, University of Minnesota, Minneapolis, MN USA; 3 BioTechnology Institute, University of Minnesota, St. Paul, MN USA; 4 Division of Colon and Rectal Surgery, Department of Surgery, University of Minnesota, 420 Delaware St. SE, Minneapolis, MN 55455 USA

**Keywords:** Colonoscopy, Large intestine, Microbiota

## Abstract

The intestinal microbiota has been implicated in the pathogenesis of complications following colorectal surgery, yet perioperative changes in gut microbiome composition are poorly understood. The objective of this study was to characterize the perioperative gut microbiome in patients undergoing colonoscopy and colorectal surgery and determine factors influencing its composition. Using Illumina amplicon sequencing coupled with targeted metabolomics, we characterized the fecal microbiota in: (A) patients (n = 15) undergoing colonoscopy who received mechanical bowel preparation, and (B) patients (n = 15) undergoing colorectal surgery who received surgical bowel preparation, composed of mechanical bowel preparation with oral antibiotics, and perioperative intravenous antibiotics. Microbiome composition was characterized before and up to six months following each intervention. Colonoscopy patients had minor shifts in bacterial community composition that recovered to baseline at a mean of 3 (1–13) days. Surgery patients demonstrated substantial shifts in bacterial composition with greater abundances of *Enterococcus*, *Lactobacillus*, and *Streptococcus.* Compositional changes persisted in the early postoperative period with recovery to baseline beginning at a mean of 31 (16–43) days. Our results support surgical bowel preparation as a factor significantly influencing gut microbial composition following colorectal surgery, while mechanical bowel preparation has little impact.

## Introduction

In 2020, an estimated 147,950 adults in the United States were diagnosed with colorectal cancer, for which the primary treatment is surgery^1^. Alternatively, diverticular disease, responsible for 200,000 inpatient admissions annually, is the leading cause of elective colon surgery, with an estimated 15% of patients eventually undergoing colectomy^2,3^. Three out of four patients with Crohn’s disease also ultimately undergo surgical resection, and half of these patient relapse and require additional surgery^4^. Thus, regardless of the disease, delivery of optimal patient care often requires bowel resection, ideally with anastomosis, that also requires an optimal environment for appropriate wound healing. However, complications, including surgical site infections (SSI), anastomotic leak, postoperative ileus (POI) and *Clostridioides difficile* (*C. difficile)* infection, following colorectal surgery remain major problems and sources of patient morbidity, mortality, and economic burden. In fact, colorectal surgery has among the highest rates of SSI, varying from 5 to 23% and a weighted mean of 11%^5^. Thus, there is an urgent need to identify strategies to prevent complications associated with colon and rectal surgery.

Postoperative complications occur despite optimal surgical technique and seemingly appropriate sterile conditions. It is likely that these complications arise from a combination of various host factors (*i.e.*, comorbidities) as well as the activity and composition of the intestinal microbiota. Once believed to be a benign bystander, the microbiota contributes to host metabolism via multiple mechanisms including energy harvest, metabolite production, and induction of pro- and anti-inflammatory immune responses^6^. The intestinal microbiota has been proposed as a primary vehicle through which host genetics and environment interact to directly affect postoperative complications. Krezalek, et al.^7^ demonstrated that mice intestinally colonized with methicillin-resistant *Staphylococcus aureus* can develop surgical site infections by migration to the wound without sepsis or bacteremia. The intestinal microbiota also plays a causative role in anastomotic leak by antagonizing wound healing through collagenase production^8^. Furthermore, the occurrence of POI has been linked to increases in the relative abundances of bacteria shown to promote pathogenic inflammation^9^.

Complications, particularly those of bacterial origin, continue to plague the field of colon and rectal surgery. The theoretical role of mechanical bowel preparation (MBP) with oral antibiotics (OA) is to facilitate the function of the host immune defense system by decreasing intestinal bacterial density and to mechanically cleanse the intestine^10^. The most commonly implemented bowel regimen includes polyethylene glycol for MBP plus neomycin and metronidazole as OA, herein the combination of MBP + OA is referred to as surgical bowel preparation (SBP)^11^. Patients undergoing colorectal resection also commonly receive perioperative intravenous (IV) antibiotics up to 24 h postoperative for additional SSI prevention.

While many studies have evaluated and compared the utility of MBP with or without OA, there is a poor understanding of the impact of these interventions on the composition of the intestinal microbiome following colorectal surgery. In this study, our primary aim was to characterize the perioperative composition of the intestinal microbiome, including short-chain fatty acids (SCFA) and fecal immunoglobulin A (IgA), of patients undergoing colorectal surgery after receiving SBP with perioperative IV antibiotics and compare these results to patients undergoing colonoscopy after receiving MBP alone. Secondary outcomes included the identification of factors associated with postoperative complications.

## Results

### Study cohorts

Fecal samples were obtained from 15 patients who underwent colonoscopy after MBP alone, and 15 patients who underwent colorectal surgery after SBP with perioperative IV antibiotics. Patient demographics and clinical data are presented in Table 1. Patients who underwent surgery were older and had greater Charlson Comorbidity Index scores^12^ compared to patients who underwent colonoscopy. Surgery patients had a median length of hospital stay of 3 (0–11) days. The colonoscopy cohort included 7 patients (47%) who underwent screening colonoscopy and 8 patients (53%) who underwent diagnostic colonoscopy for hematochezia (n = 7) or chronic diarrhea (n = 1). The surgery cohort included 11 patients (73%) who underwent colorectal resection for colorectal cancer (n = 6) or diverticular disease (n = 5), and 4 patients (27%) who underwent non-resectional colorectal surgery for rectal prolapse (n = 2) or rectal neoplasia (n = 2). Operative details and clinical history of surgery patients are detailed in Table 2.

**Table 1:** Patient demographic and clinical data.

	Colonoscopy (n = 15)	Surgery (n = 15)	*P*-value
Female sex, n (%)	10 (67)	7 (47)	0.269
Age (years)	48.3 ± 15.3	56.9 ± 11.4	0.038
BMI (kg/m^2^)	24.4 ± 5.6	27.7 ± 6.0	0.068
Albumin	3.8 ± 0.6	3.9 ± 0.4	0.945
Charlson comorbidity index	0.33 ± 0.9	1.27 ± 1.2	0.007
**Smoking status, n (%)**			0.243
Active smoker	1 (7)	4 (27)	–
Former smoker	3 (20)	4 (27)	–
Non-smoker	11 (73)	7 (46)	–
Chronic narcotics, n (%)	0	1 (7)	0.309
**History of diabetes mellitus, n (%)**	0	2 (13)	0.143
Metformin	–	1 (7)	–
**Pre-operative bowel prep, n (%)**			0.219
Miralax and Mag Citrate	14 (93)	13 (87)	–
GoLYTELY	1 (7)	0	–
Fleet enemas	0	2 (13)	–
**Post-operative complications, n (%)**			0.143
None	15	13 (86)	–
Anastomotic leak/Sepsis	–	1 (7)	–
Urinary tract infection	–	1 (7)	–
Surgical site infection	–	–	–
Deep organ space infection	–	–	–
Pneumonia	–	–	–
Ileus requiring nasogastric tube	–	–	–
Anemia requiring transfusion	–	–	–
Acute renal failure	–	1 (7)	–
Deep venous thrombosis	–	1 (7)	–
Mean length of hospital stay	0	3.1 ± 2.8	< 0.001
30-day hospital readmission, n (%)	1 (7)	3 (20)	0.283

Continuous data presented as mean ± standard deviation. *BMI* body mass index.

**Table 2:** Perioperative details and clinical history of surgery patients.

	n (%)
**Pre-operative enteral antibiotics**	
Neomycin plus Metronidazole	15 (100)
Perioperative intravenous antibiotics	
Ciprofloxacin plus Metronidazole	10 (67)
Trimethoprim/sulfamethoxazole plus Metronidazole	1 (7)
Cefotetan	4 (26)
**Operative procedure**	
Right colectomy	5 (33)
Left colectomy	1 (7)
Sigmoid colectomy	3 (20)
Sigmoid colectomy, diverting loop ileostomy	1 (7)
Low anterior resection	1 (7)
Ventral rectopexy	2 (13)
Transanal excision	2 (13)
History of colorectal cancer	6 (40)
**Pathology**	
Adenocarcinoma	5 (33)
Neuroendocrine tumor	1 (7)
**Stage**	
I	3 (20)
IIA	1 (7)
IIIA	2 (13)
History of diverticulitis	5 (33)
**Type**	
Recurrent, uncomplicated	4 (27)
Complicated (rectovaginal fistula)	1 (7)

### Microbial community composition and diversity

**Figure 1:**
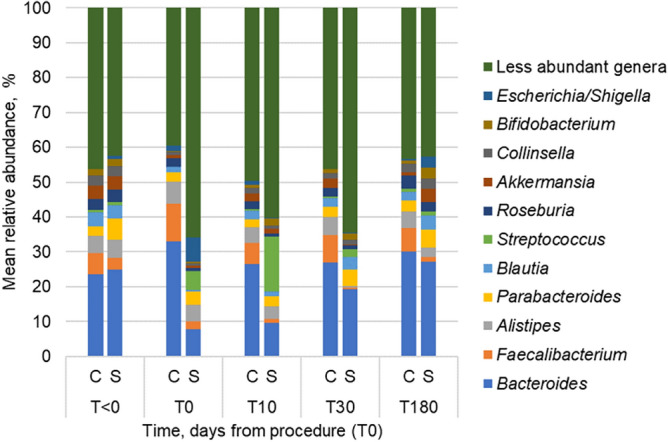
Distribution of abundant genera in patient fecal samples. Genus-level classification of predominant OTUs among patients that underwent colonoscopy (c) and surgery (s) over study course.

We performed 16S rRNA gene-based amplicon sequencing to characterize the taxonomic profiles of gut microbiota in pre-, intra-, and post-procedural or postoperative samples. Among all patients, the most abundant genera of bacteria included *Bacteroides*, *Faecalibacterium*, *Alistipes*, *Parabacteroides*, *Blautia*, *Streptococcus*, and *Roseburia* (Fig. 1). Significant changes in Shannon indices were not observed in samples from patients undergoing colonoscopy, while samples from surgical patients had significantly lower alpha diversity immediately following surgery (Supplemental Table S2; Tukey’s *post-hoc P* < 0.001). Among specimens from patients undergoing surgery, there were no significant difference in Shannon indices between resectional and non-resectional colorectal surgery patients over the study course (*P* = 0.142).

**Figure 2:**
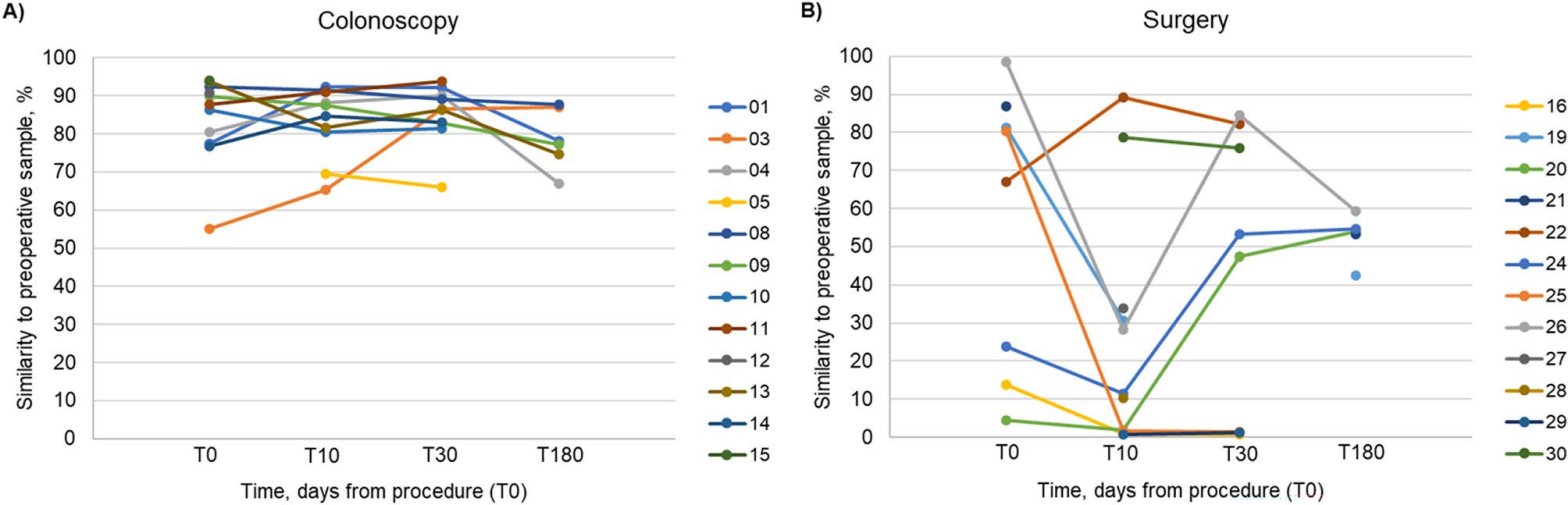
Change in microbial composition over study course as determined by SourceTracker. Percent similarity of intra- and postoperative samples of individual patients to their own preoperative sample among patients that underwent (**A**) colonoscopy and (**B**) surgery.

To assess the change in gut microbial composition in individual patients over the study course, we used SourceTracker2 software, and found that patients who underwent colonoscopy did not have significant changes in their overall microbial composition from that observed pre procedure (Fig. 2A). However, surgery patients experienced a substantial change in gut microbial composition within the early postoperative period up to 10 days (Fig. 2B). At T_10_, samples from nine surgery patients (60%) had < 35% similarity to pre-operative microbial community composition, of which four patients (27%) had < 2% similarity. Samples from two surgery patients were > 75% similar to pre-operative microbial composition. Patients within the surgery cohort experienced recovery of preoperative microbiota at varying frequencies. Three of nine patients (33%) with extreme early postoperative changes in gut microbial composition began to repopulate microbial communities by one month (T_30_), while compositional changes in three of these nine patients persisted at this timepoint.

### Effects of OA in conjunction with MBP

**Figure 3:**
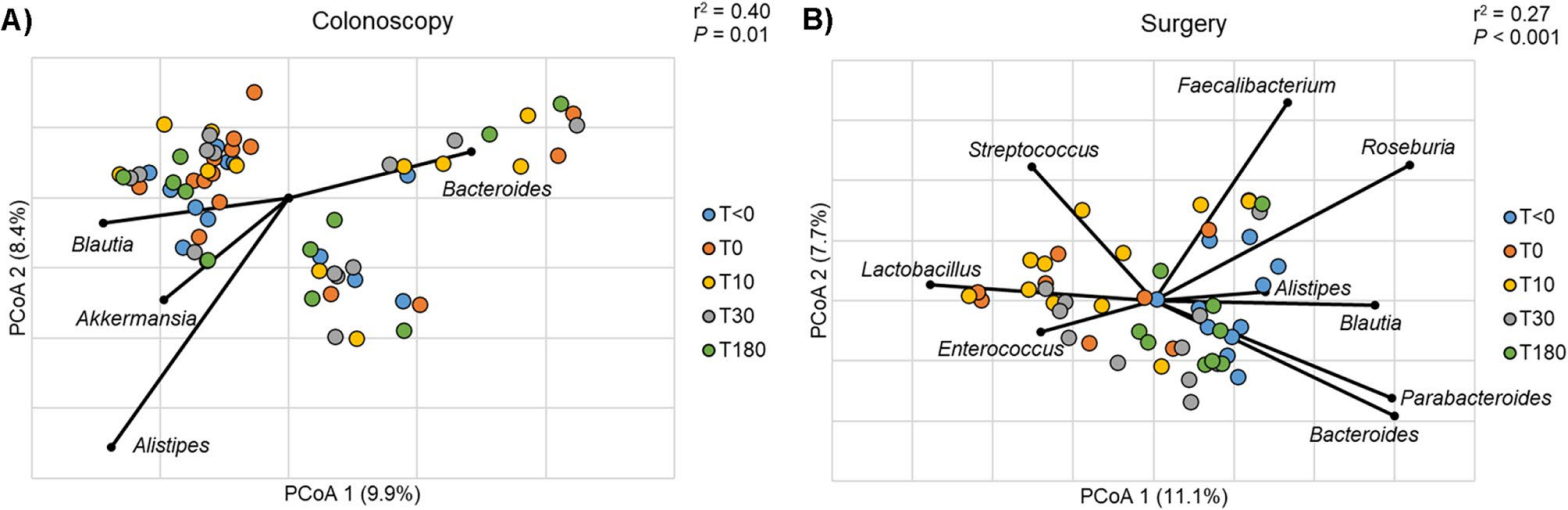
Principle coordinate analysis of Bray–Curtis distances among samples collected over study course in patients that underwent (**A**) colonoscopy and (**B**) surgery. Abundant genera significantly correlated to axis positions by Spearman correlation are shown (P < 0.05).

Colonoscopy and surgery cohorts had significantly different bacterial community compositions at baseline (Fig. 3; ANOSIM, R = 0.13, *P* = 0.016), and were analyzed separately because of this. While no longitudinal changes in community composition were observed among patients who underwent colonoscopy (Fig. 3A), MBP alone significantly changed community composition intra-procedure (T_0_), which was dissimilar from pre-procedural (T_<0_) and late post-procedural (T_30_ and T_180_) samples (Table 3; ANOSIM, R = 0.17–0.27, *P* = 0.001–0.004). In contrast, communities from surgery patients showed a directional shift away from the preoperative assemblage in both the intra- and short-term postoperative samples (T_0_ and T_10_), which had greater abundances of *Enterococcus*, *Lactobacillus*, and *Streptococcus* (Fig. 3B). Samples in the intra- and short-term postoperative period (T_10_) showed significant dissimilarity from both preoperative (T_<0_) and late postoperative (T_30_ and T_180_) samples (Table 3; ANOSIM, R = 0.26–0.47, *P* = 0.001–0.004). Increased abundances of genera within the Firmicutes and Bacteroidetes phyla were observed in both preoperative (T_<0_) and late (T_30_ and T_180_) postoperative samples (Fig. 3B).

**Table 3:** ANOSIM comparison of Bray–Curtis dissimilarities.

	(A) Colonoscopy				(B) Surgery			
	T < 0	T0	T10	T30	T < 0	T0	T10	T30
T0	**0.27, < 0.001**				**0.47, < 0.001**			
T10	0.01, 0.382	0.10, 0.053			**0.35, < 0.001**	0.02, 0.37		
T30	− 0.02, 0.696	**0.17, 0.003**	− 0.02, 0.63		**0.21, 0.002**	**0.29, 0.005**	**0.26, 0.002**	
T180	− 0.01, 0.587	**0.22, 0.004**	− 0.06, 0.934	− 0.09, 0.997	0.11, 0.069	**0.33, < 0.001**	**0.26, 0.004**	0.03, 0.308

ANOSIM R-values and *P* values are presented in the matrix for (A) colonoscopy and (B) surgical samples. Bold indicates significant *P* value (< 0.05).

To further describe the differences in microbial composition between colonoscopy and surgery patients over the study course, we performed an analysis with splinectomeR (Supplemental Fig. S2). Over the study period, there were significant reductions in the relative abundances of *Bacteroides*, *Faecalibacterium*, and *Roseburia* in surgery patients compared to colonoscopy patients (*P* = 0.001). Furthermore, surgery patients had a significant increase in the relative abundance of *Streptococcus* compared to colonoscopy patients (*P* < 0.001).

### Single patient with anastomotic leak

One patient, who underwent robotic low anterior resection for rectal adenocarcinoma, presented with postoperative anastomotic leak on postoperative day five, after discharging from the hospital. This patient was taken urgently to the operating room for pelvic washout, primary repair of the anastomotic defect and diverting loop ileostomy. Fecal samples from this patient showed < 1% similarity to preoperative microbial composition in the short-term post-operative period (T_10_; collected postoperative day 1), as determined by SourceTracker2, which persisted at one month (T_30_). Approximately five months after index operation, the patient underwent ileostomy reversal after receiving perioperative IV antibiotics without MBP or OA. A sample was collected at this time intraoperatively from the unprepped colon. This was observed to have 56% similarity to the initial preoperative sample and showed a shift toward the preoperative assemblage on PCoA (Supplemental Fig. S3). The microbial community in a subsequent sample, obtained on postoperative day one after ileostomy reversal, was 1.2% similar to preoperative composition by SourceTracker and resembled those of other early postoperative samples. A final sample, obtained six months after ileostomy reversal, was 63% similar to the initial preoperative sample and appeared to be similar to other 6-month post-operative samples.

### Evaluation of short-chain fatty acids

Preoperative, T_10_, and T_30_ samples were further analyzed for SCFA composition (Table 4). Due to insufficient sample volume, intra-operative samples were excluded from analysis. Among T_10_ samples, one sample in the colonoscopy group and eight in the surgery group had SCFA concentrations within the range observed in the blanks (within the mean ± 1 standard deviation) and were excluded from statistical comparisons, as were two surgery samples at the T_30_ time point. Differences in detection of SCFA between colonoscopy and surgery groups did not differ significantly at any time point (χ^2^ *P* = 0.218). Among all time points, concentrations of butyric acid and valeric acid were significantly greater in the colonoscopy group (*P* = 0.010 and 0.031, respectively), but no other significant differences were observed. Subsequent longitudinal analysis using SplinectomeR indicated that the greatest difference in concentrations between treatment groups occurred at the T_10_ time point (Supplemental Fig. S4). Among the colonoscopy samples, concentrations of SCFA predominantly correlated with relative abundances of *Alistipes* and *Akkermansia* (Supplemental Table S3). Conversely, among the surgical samples, SCFA concentrations correlated with abundances of *Parabacteroides* and *Blautia* and negatively correlated with *Streptococcus*.

**Table 4:** Concentrations as mean (SD) of SCFA (μM).

Procedure	Time point (n)	Acetic	Propionic	Isobutyric	Butyric	Isovaleric	Valeric	4-Methylvaleric
Colonoscopy	T_<0_ (10)	753.6 (275.4)	500.8 (226.7)	86.7 (52.5)	469.4 (282.4)	67.7 (51.8)	91.4 (55.5)	2.6 (1.5)
	T_10_ (10)	732.1 (265.2)	459.8 (293.0)	71.8 (61.5)	355.8 (185.0)	55.4 (53.7)	69.4 (61.9)	2.1 (0.8)
	T_30_ (10)	758.4 (369.0)	469.3 (163.0)	57.6 (41.2)	448.1 (239.0)	46.5 (44.7)	67.2 (47.2)	3.0 (2.2)
Surgery	T_<0_ (10)	839.5 (298.2)	516.0 (188.7)	59.3 (37.4)	375.7 (192.7)	46.4 (32.3)	63.2 (50.2)	1.9 (0.5)
	T_10_ (10)	116.8 (178.6)	62.1 (104.4)	6.9 (14.6)	88.3 (20.3)	8.2 (17.5)	1.0 (1.1)	1.4 (0.3)
	T_30_ (10)	513.6 (495.0)	254.8 (256.2)	34.2 (27.2)	199.3 (135.5)	27.0 (23.6)	4.6 (4.0)	3.7 (5.9)
Blanks (6)		13.0 (21.1)	18.1 (0.2)	0.1 (0.2)	79.3 (0.0)	0.5 (0.2)	0.5 (0.2)	1.2 (0.2)

### Fecal IgA and calprotectin

Although fecal IgA levels did not significantly change from preoperative (T < 0) to early postoperative (T_10_) samples among patients that underwent either colonoscopy or colorectal surgery (Supplemental Fig. S5; Tukey’s *post-hoc P* = 0.415), we found that concentrations of fecal IgA inversely correlated with microbial diversity by Shannon index (Spearman *ρ* = −0.69, *P* < 0.0001). Fecal calprotectin levels also did not significantly change from preoperative (T_<0_) to early postoperative (T_10_) samples among patients who underwent colonoscopy or colorectal surgery (Supplemental Fig. S6; *P* = 0.134).

## Discussion

The composition of the intestinal microbiome appears to be a major contributor to the occurrence of postoperative complications including SSI, anastomotic leak, POI, and *C. difficile* infection^6,8,9,13^. However, the impact of important perioperative factors that are known to shape its composition have not been described. This study showed that while MBP alone does change the composition of the microbiome, the magnitude of this shift is small, and the gut microbiome composition reverts to baseline by ten days following colonoscopy. In contrast, patients who received SBP displayed acute shifts of greater magnitude, requiring at least 30 days to return to a composition similar to baseline. Colorectal surgery with or without bowel resection did not appear to significantly impact the gut microbiome among surgical patients, as changes were observed in all such patients, independent from underlying disease or type of colorectal resection. While the aim of our study was to assess perioperative shifts in the microbiota, our sample sizes and patient heterogeneity exclude specific conclusions related to type or surgery, disease, or patient outcome. In this study, MBP with OA was the single most influential factor in shaping the composition of the gut microbiome acutely and longitudinally following colon and rectal surgery.

These observations build on our previous work, that perioperative antibiotics most significantly impact acute postoperative changes in the microbiome, to a greater extent than caloric restriction or gastric resection, following vertical sleeve gastrectomy^14^. The impact of SBP is demonstrated longitudinally in the gut microbial composition with potential implications for postoperative complications in patients undergoing colon and rectal surgery. Communities from surgery patients showed a directional shift away from preoperative assemblages, with greater abundances of *Enterococcus*, *Lactococcus*, and *Streptococcus.* These changes are noteworthy as they may be associated with adverse post-operative outcomes. For example, *Enterococcus* has been mechanistically linked to the development of anastomotic leak via the production of matrix metalloproteinase 9, which degrades collagen leading to tissue breakdown^8^. *Enterococcus* and *Streptococcus* are also potent bacteriocin and lactic acid producing bacteria^15,16^, and may be general markers for dysbiotic stress in the clinical setting. These bacteria dominate the microbiota composition, disrupt the gut barrier, and are associated with the subsequent development of bacteremia in patients who received antibiotics for allogeneic hematopoietic stem cell transplantation (allo-HSCT). Patients who undergo allo-HSCT have shown to develop markedly reduced microbial diversity which is an independent predictor of mortality^17^. In fact, patients who are dominated by these particular organisms are at highest risk for bloodstream infections during allo-HCST^18^. Overall, our data are consistent with many of the deleterious changes observed in patients undergoing allo-HSCT.

We also observed a significant reduction in the relative abundance of critical genera including *Bacteroides*, *Faecalibacterium*, and *Roseburia* in surgery patients compared to colonoscopy patients. Anastomotic leak has been associated with a relative paucity of *Faecalibacterium*^19^. Abundances of both *Faecalibacterium* and *Roseburia* genera strongly correlate with microbial diversity, and their reduction has been suggested in the pathogenesis of ulcerative colitis^19–21^. Interestingly, we noted correlations between SCFA and butyrate producing *Roseburia* and *Blautia* among samples collected in the surgical arm, while *Faecalibacterium* negatively correlated with SCFA concentrations among patients undergoing colonoscopy. These results suggest that typically cited butyrate producers may act in a compensatory manner following surgery to promote healing, while normal microbial communities shift this function to members of Bacteroidetes (e.g., *Alistipes*), as observed in the colonoscopy group. This suggestion further highlights the need to explore microbial community interactions to better assess the role of the microbiome in postoperative healing and complications. It is noteworthy that fecal levels of butyric acid and valeric acid precipitously declined following surgery. Dysbiotic microbiota with depleted SCFA results in dysregulated immune function propagating intestinal and systemic inflammation and colitis impeding wound healing^22^. This likely has greater significance in the immediate postoperative phase in which colonic healing is actively taking place at a time when butyric acid and valeric acid are at their nadir levels.

Our singular anastomotic leak patient provides an effective case study for the effects of SBP. Preoperatively, the patient’s microbial composition was similar to that of the surgical cohort, and while the microbial composition shifted with MBP plus OA, the anastomotic leak patient’s composition was overwhelmingly dominated by *Streptococcus*. Notably, postoperative IgA levels were 16-fold higher in this patient compared to the perioperative sample, suggesting a significant and concomitant increase in gut inflammation. This patient displayed significant shifts in composition after each surgery, highlighting the impacts of SBP and perioperative IV antibiotics on the intestinal microbiome. This patient also highlights a potentially deleterious impact of SBP, and perhaps instead of protecting the patient, providing an ecologic advantage for streptococcal propagation of inflammation and anastomotic breakdown.

Our study does have important weaknesses, including the lack of mucosal microbiota, which may be different from the luminal microbiome reflected in stool^23,24^. Secondly, a larger cohort of patients is required to verify our observations. This would also enable the identification of more specific changes in the microbiome that correlate with postoperative outcomes. Additionally, we included patients undergoing a variety of different types of operations. While this broad inclusion makes it difficult to identify a relationship between SBP and compositional changes in the gut microbiome, it does strengthen one of the findings of this study—that type of colorectal surgery with or without bowel resection impacts the composition of the microbiome to a lesser extent than the profound impact of SBP. Also, while we did not observe a change in fecal IgA or calprotectin, we did identify a strong inverse correlation between IgA levels and alpha diversity, which is consistent with previous reports^25^. It remains possible that IgA levels and fecal calprotectin do change with postoperative shifts in the microbiome, but a larger cohort is required to investigate potential relationships. Future studies with more patients are required to answer these questions which were beyond the scope of this study.

In conclusion, this study showed that SBP with perioperative IV antibiotics has a significant impact on the postoperative composition of the gut microbiome following colon and rectal surgery. Although SBP represents a critical tool that has been associated with less intestinal microbial-driven complications, this mechanism of action via the microbiome remains unclear and requires further investigation. While effective, SBP remains a shotgun approach that does not consider the patient’s native microbiome, thus hindering a personalized approach to optimizing patient outcomes following surgery. The eradication of the intestinal microbiome acutely following surgery may represent a unique opportunity to intervene and sets forth a premise for the re-introduction of critical commensal bacteria that contribute to gut health and recovery at a time when the patient needs it most.

## Methods

### Patient cohort

A total of 30 adult patients were recruited from March 2019 through October 2020 and underwent either colonoscopy (n = 15) or elective colorectal surgery (n = 15) at the University of Minnesota Medical Center. Fecal samples were obtained pre-procedural or preoperatively (T_<0_, mean 6 [1–33] days), intra-procedural or intraoperative (T_0_), post-procedure or postoperative within 10 days (T_10_, mean 3 [1–13] days), within 3–6 weeks (T_30_, mean 31 [16–43] days), and at 6 months (T_180_, mean 199 [188–246] days). Colonoscopy patients underwent MBP alone, while surgery patients underwent SBP composed of MBP with OA and 24 h of postoperative IV antibiotics. MBP included Miralax (238 g) plus Magnesium Citrate (296 mL; n = 27), GoLYTELY (236 g; n = 1), or two fleet enemas (n = 2). Oral antibiotics included neomycin (1 g q6h for 3 doses) plus metronidazole (500 mg q6h for 3 doses; n = 15), given with prophylactic Zofran (4 mg q6h for 3 doses) as an anti-nausea measure, given our previous experience with frequent nausea and vomiting thought to be caused by oral metronidazole. Perioperative IV antibiotics were initiated 1–2 h before incision and continued for up to 24 h postoperative and included ciprofloxacin (400 mg q12h) plus metronidazole (500 mg q8h; n = 10), trimethoprim/sulfamethoxazole (800–160 mg q24h) plus metronidazole (500 mg q8h; n = 1), and cefotetan (1–2 g q12h; n = 4) at the discretion of the surgeon. Diet is commonly restricted in patients following surgery. Post-operative diet consisted of clear liquids until evidence of return of bowel function which occurred between one to three days following surgery. Patients were then advanced to a low residue, low fiber diet which was continued for six weeks at which time dietary restrictions were lifted. Exclusion criteria included history of solid or liquid organ transplant, active immunosuppressive medication, chronic steroid use, any chemotherapy 12 months before colonoscopy or surgery, history of intra-abdominal or pelvic radiation, history of inflammatory bowel disease, history of previous colorectal resection, use of any antibiotic 30 days before enrollment, planned diverting ileostomy, and pregnancy. Human fecal specimens were obtained under approval by the Institutional Review Board of the University of Minnesota. Informed written consent was obtained from each study participant before enrollment.

### Sample collection

Fecal samples were collected by patients in single-use specimen collector pans and transferred to 30 mL polystyrene fecal specimen containers (Globe Scientific, Inc., Paramus, NJ, USA). Patients stored samples in their home freezers prior to their clinic, procedure, or surgery visit, when samples were transferred to a − 80 °C freezer until use. Intra-procedural or intraoperative samples were collected by the treating surgeon via rigid proctoscopy, flexible sigmoidoscopy, or colonoscopy via endoscopic suction of colonic effluent from the rectum and sigmoid colon with as needed lavage using sterile water or saline solution in cases where no effluent was present in the colon or rectum. Specimens were placed in 30 mL polystyrene fecal specimen containers and immediately transferred to a − 80 °C freezer until processing.

### DNA extraction and sequencing

DNA was extracted from 250 mg thawed fecal samples using the DNeasy PowerSoil kit (QIAGEN, Hilden, Germany) on the automated QIAcube platform following the inhibitor removal technology (IRT) protocol. The V4 hypervariable region of the 16S rRNA gene was amplified using the 515F/806R primer set^26^ by the University of Minnesota Genomics Center (UMGC, Minneapolis, MN, USA), as previously described^27^. Paired-end, dual-indexed sequencing at a read length of 300 nucleotides (nt) was performed on the Illumina MiSeq platform (Illumina, Inc., San Diego, CA, USA) by UMGC^27^. Negative sterile water controls were included in all sequencing runs and did not produce amplicons. Raw data were stored in the Sequence Read Archive under BioProject accession number SRP250717.

### Bioinformatics

Amplicon sequence data were processed and analyzed using mothur software ver. 1.41.1^28^ with our previously published pipeline for quality screening and taxonomic annotation^29^. Briefly, reads were paired-end joined, quality trimmed, and aligned against the SILVA database ver. 138^30^. Sequences were further cleaned using a 2% pre-clustering step^31^ and chimeric sequences were identified and removed using UCHIMER ver. 4.2.40^32^. Operational taxonomic units (OTUs) were binned at 99% similarity using the furthest-neighbor algorithm and taxonomic assignment was done against the Ribosomal Database Project ver. 16^33^. A mean (± standard deviation) of 18,058 ± 10,916 reads per sample were obtained. For unbiased statistical comparisons^34^, samples were rarefied to 4,500 sequence reads by random subsampling, and samples with fewer sequence reads were removed from the data set. After rarefaction, a mean estimated Good’s coverage of 98.14 ± 0.02% and a mean of 187 ± 90 OTUs were observed among all samples. The number of fecal samples included in comparisons among sample groups at each time point after normalization by rarefaction are shown in Fig. 1.

Sourcetracker2 was used with default parameters to assess the similarity of intra- and post-operative microbial composition of individual patients to their own pre-operative microbiota^35^. This software uses a Bayesian inference approach to determine what percent of the community of sink samples (intra- and post-operative samples) is derived from source (pre-operative) samples taking an OUT table as input.

### Statistical analysis

A power calculation was done using the MicroPower package in R^36^. This package models OTU tables based on user-defined inputs and determines power to detect differences when using a PERMANOVA test. Communities were modeled based on study data from a pool of 5,000 OTUs among all samples, with 200 OTUs per sample and 25% OTU bin retention. Our sample size powered detection of a small effect size (*ω*^2^ = 0.03) at 1 − *β* = 1.00 and *α* = 0.05.

Alpha and beta diversity statistics were calculated in mothur. Alpha diversity was evaluated with the Shannon index^37^, which accounts for richness and evenness. Beta diversity was calculated using Bray–Curtis distances^38^ and was visualized by ordination using principal coordinate analysis (PCoA)^39^. Spearman correlations were calculated to determine abundant genera that were significantly associated with PCoA axis position using the corr.axes command in mothur. Differences in taxon abundances over the course of the study were determined using the *permuspliner* function of SplinectomeR^40^, a permutation-based package in R that uses weighted local polynomials to test for group differences in longitudinal microbiome data, with multiple time points per subject. The *sliding_spliner* function of SplinectomeR, a technique that divides the time axis into 100 segments and finds segments with larger contributions to the overall intergroup difference over time for a given taxon, was used to determine whether groups were significantly different at specific time points.

Patient demographic and clinical data were compared using Chi-squared analysis for categorical variables and Mann–Whitney-U test for continuous variables. Differences in Shannon index and SourceTracker analysis were determined using ANOVA with Tukey’s *post-hoc* test. Community compositions at different time points were compared using analysis of similarity (ANOSIM)^41^ with Bonferroni correction for pairwise comparisons. Analyses were conducted with XLSTAT (version 2020.2.3; Addinsoft, Belmont, MA, USA). All statistics were evaluated at *α* = 0.05.

### Ethics approval

Approval for this study was given by the University of Minnesota Institutional Review Board (Protocol Number: 00005429). All subjects provided written informed consent for participation in the study and collection and analysis of data. All study procedures were in accordance with the ethical standards of the national research ethics committee and with the 1964 Helsinki declaration and its later amendments or comparable ethical standards.

## Supplementary Information

Supplementary Information.

## Data Availability

Sequence data are deposited in the Sequence Read Archive under BioProject accession number SRP250717. Available at: https://www.ncbi.nlm.nih.gov/sra/?term=SRP250717.

## Abbreviations

SSI: Surgical site infections
POI: Postoperative ileus
*C. difficile*: *Clostridioides difficile*
MBP: Mechanical bowel preparation
OA: Oral antibiotics
SBP: Surgical bowel preparation
IV: Intravenous
SCFA: Short-chain fatty acids
IgA: Immunoglobulin A
