# Supplementary material for: A pilot study demonstrating the impact of surgical bowel preparation on intestinal microbiota composition following colon and rectal surgery
Source: Sci Rep. 2022 Jun 22;12:10559. doi: 10.1038/s41598-022-14819-1 (PMC9217797; doi:10.1038/s41598-022-14819-1)
Supplement: Supplementary file 1 — Supplementary Information. [file 41598_2022_14819_MOESM1_ESM.docx]

**
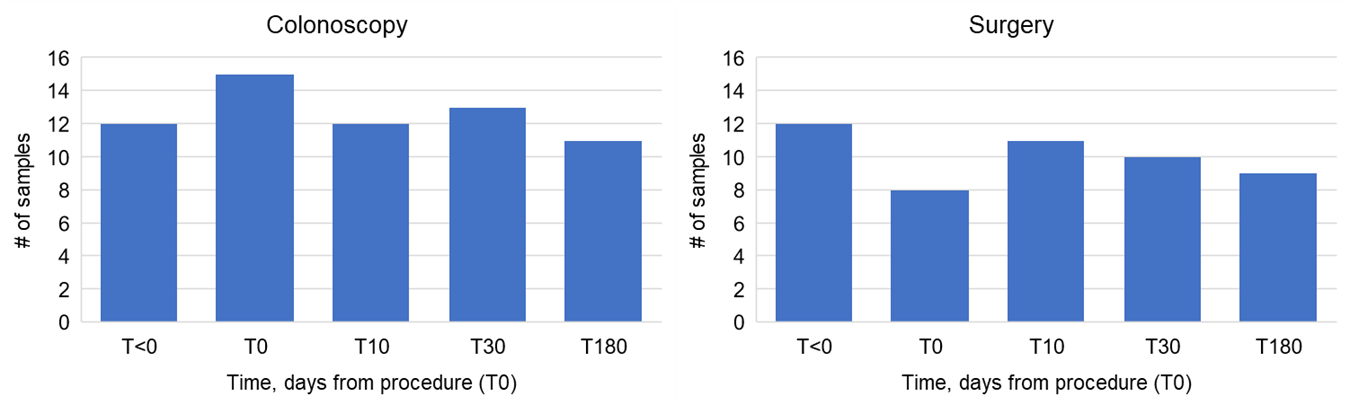
**

**Supplemental Figure 1.** Number of fecal samples included in comparisons among sample groups by patients that underwent A) colonoscopy and B) surgery, after sample normalization by rarefaction.

**
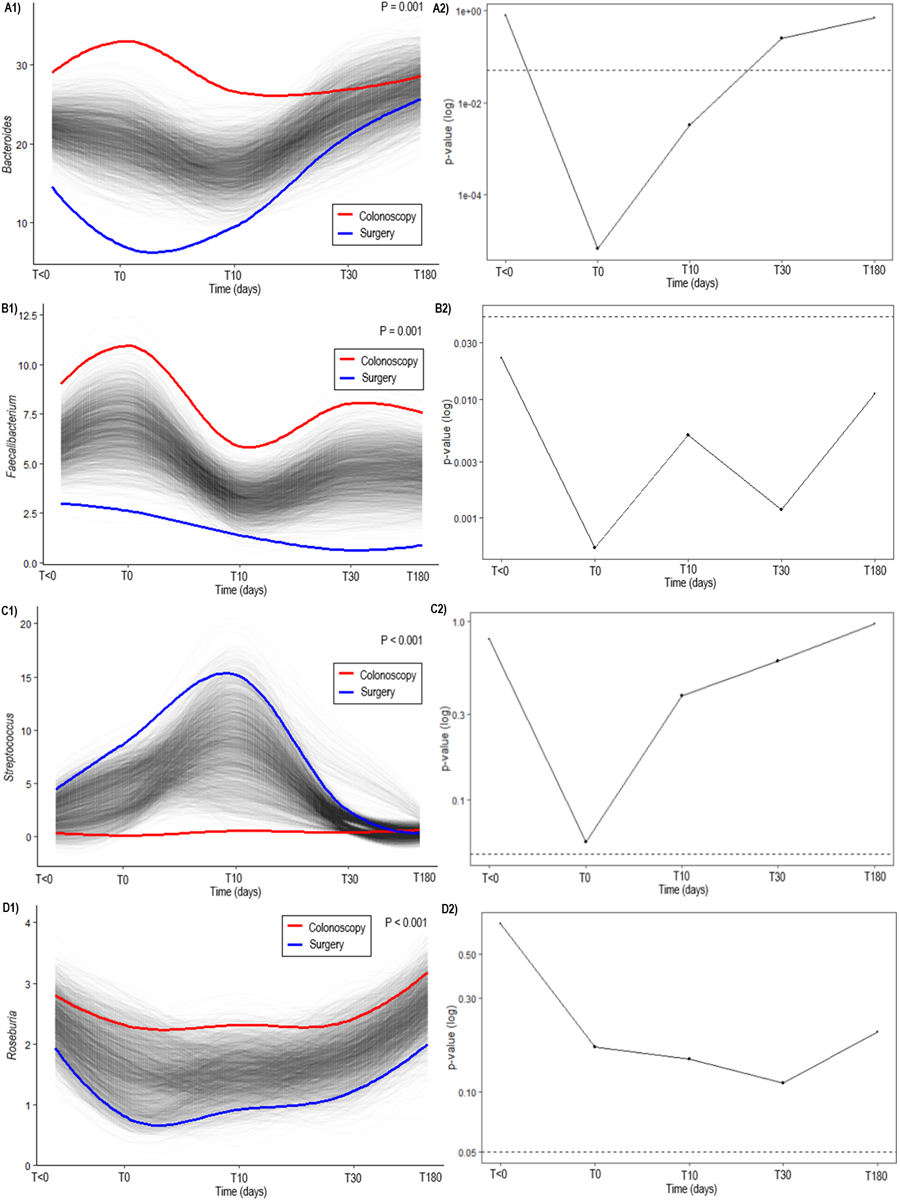
**

**Supplemental Figure 2.**  Permuted spline tests for statistical significance in longitudinal microbial data shows differences between patients undergoing colonoscopy and surgery in relative abundance of A) *Bacteroides*, B) *Faecalibacterium*, C) *Streptococcus*, and D) *Roseburia*.

**
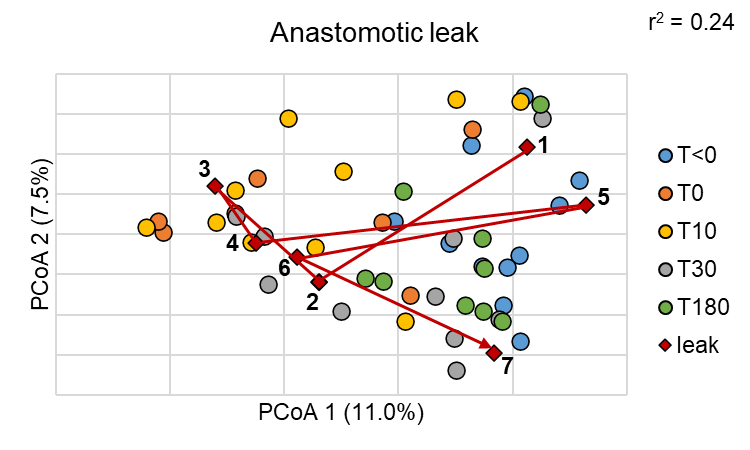
**

**Supplemental Figure 3.** Principle coordinate analysis of Bray-Curtis distances among samples collected over study course in patients that underwent surgery, including patient who had postoperative anastomotic leak (red). Samples 1 (T<0), 2 (T0), 3 (T1), 4 (T29), 5 (T153), 6 (T154), and 7 (T335) are shown.


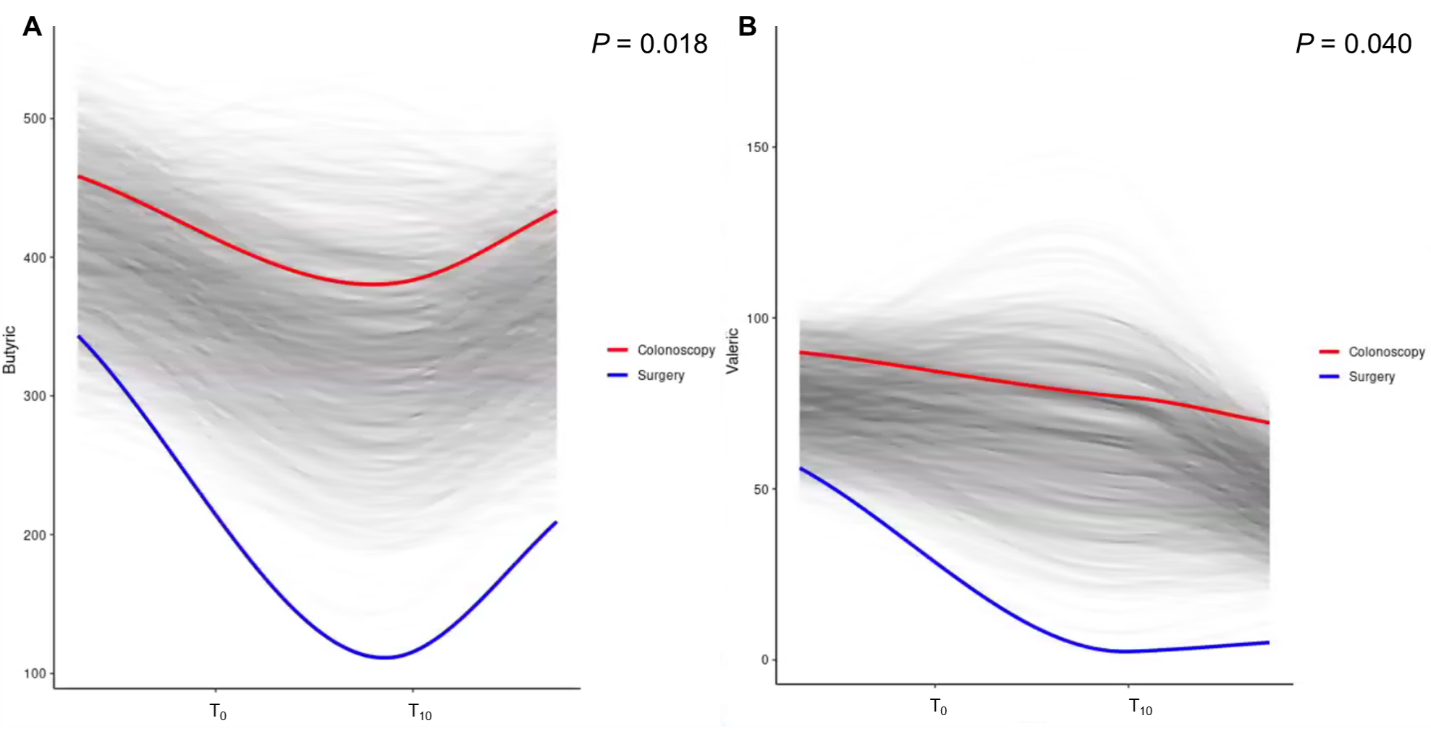
 **Supplemental Figure 4.**  Longitudinal analysis of SCFA concentrations. A) Butyric acid and B) valeric acid analyzed using SplinectomeR.


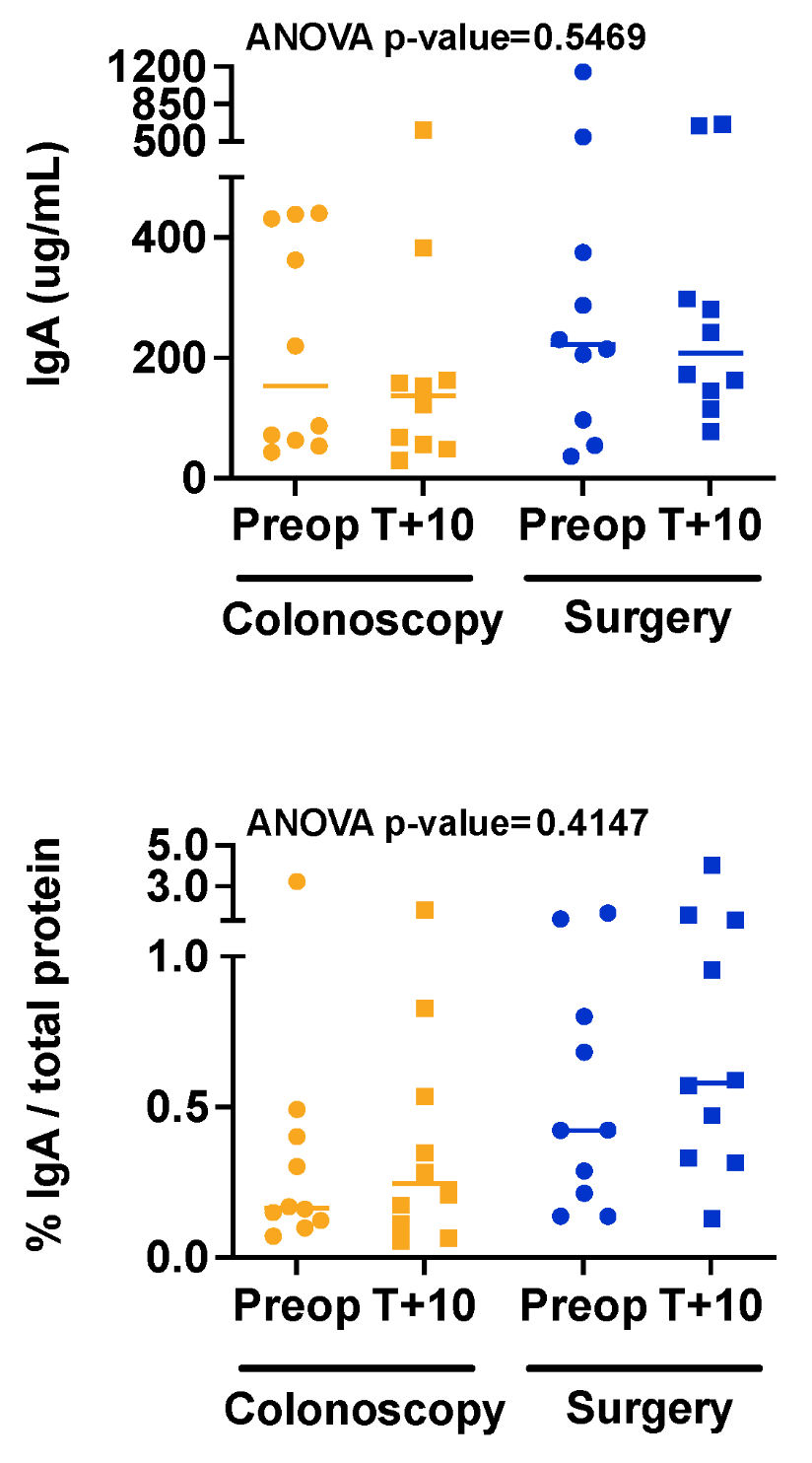


**Supplemental Figure 5.** (A) Fecal IgA concentrations and (B) Percent Fecal IgA concentrations relative to total protein prior to and acutely (T10) following colonoscopy or surgery.


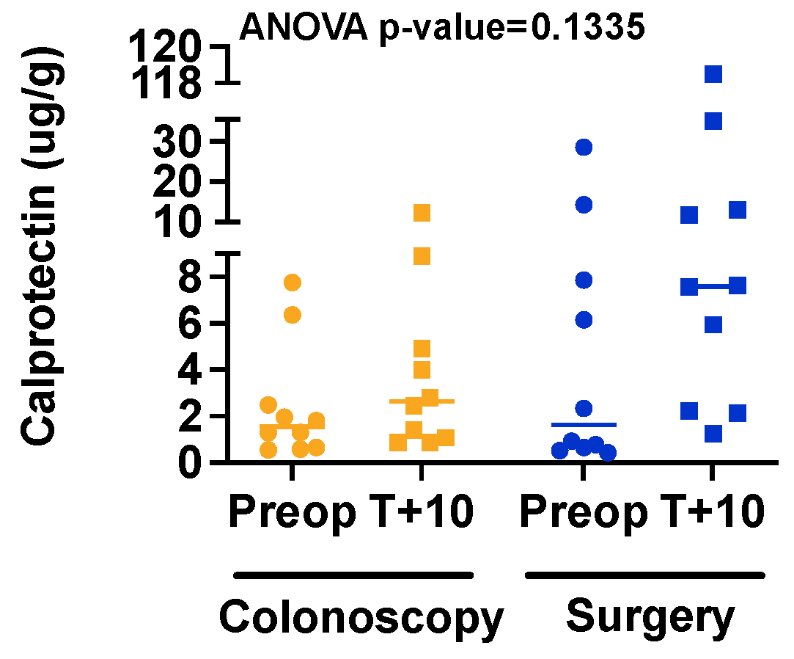


**Supplemental Figure 6.** Fecal calprotectin concentrations prior to and acutely (T10) following colonoscopy or surgery.

**Supplemental Table 1.** Computational parameters and curve fit for SCFA analysis.

| Compound | Quant Ion (m/z) | Expected RT (min) | Cal Curve | R^2^ |
| --- | --- | --- | --- | --- |
| Acetic Acid | 43.0185 | 5.339 | Y = 0.004861 * x + 1.976720 | 0.9939 |
| Propionic Acid | 73.0286 | 5.966 | Y = 0.005784 * x + 0.9972377 | 0.9972 |
| Isobutyric Acid | 73.0289 | 6.200 | Y = 0.009044 * x + 0.016972 | 0.9990 |
| Butyric Acid | 55.0180 | 6.715 | Y = 0.005401 * x - 0.428140 | 0.9918 |
| Isovaleric Acid | 60.0210 | 7.107 | Y = 0.025248 * x + 0.028839 | 0.9983 |
| Valeric Acid | 60.0210 | 7.795 | Y = 0.026160 * x – 0.007053 | 0.9982 |
| 2-Ethylbutyric Acid | 88.0523 | 8.060 |  |  |
| 4-Methylvaleric Acid | 57.0703 | 8.503 | Y = 0.012271 * x – 0.011853 | 0.9956 |

**Supplemental Table 2.** Shannon indices (mean ± standard deviation) of fecal communities from patients undergoing colonoscopy and surgery. Time represented as days from procedure (T0). Samples sharing the same letter did not vary significantly by Tukey's *post-hoc* test (*P*<0.001).

| **Time (days)** | **Colonoscopy** | **Surgery** |
| --- | --- | --- |
| T<0 | 3.8 ± 0.3 A | 3.6 ± 0.4 A |
| T0 | 3.2 ± 0.4 AB | 3.4 ± 0.4 AB |
| T10 | 3.4 ± 0.7 AB | 2.8 ± 1.1 B |
| T30 | 3.7 ± 0.3 A | 3.1 ± 0.4 AB |
| T180 | 3.8 ± 0.4 A | 3.4 ± 0.3 AB |

**Supplemental Table 3.** Spearman correlations relating SCFA concentrations to relative abundances of predominant genera. Values in parentheses are Spearman’s rho; *P*-values. Samples from all time points were used for each treatment group. No significant correlations were observed with 4-methylvaleric acid.

| **SCFA** | **Colonoscopy** | **Surgery** |
| --- | --- | --- |
| Acetic | *Alistipes* (-0.381; 0.039)  *Collinsella* (0.363; 0.049) | *Parabacteroides* (0.420; 0.038)  *Blautia* (0.742; < 0.0001)  *Streptococcus* (-0.564; 0.004) |
| Propionic | *Faecalibacterium* (-0.385; 0.037) | *Bacteroides* (0.402; 0.047)  *Parabacteroides* (0.657; < 0.0001)  *Blautia* (0.552; 0.005)  *Streptococcus* (-0.435; 0.031) |
| Isobutyric | *Faecalibacterium* (-0.426; 0.020)  *Alistipes* (0.482; 0.008)  *Akkermansia* (0.478; 0.008) | *Parabacteroides* (0.417; 0.039)  *Blautia* (0.440; 0.029) |
| Butyric | *Roseburia* (0.373; 0.043)  *Collinsella* (0.402; 0.028) | *Parabacteroides* (0.424; 0.036)  *Blautia* (0.608; 0.002)  *Streptococcus* (-0.488; 0.014)  *Roseburia* (0.49; 0.014) |
| Isovaleric | *Faecalibacterium* (-0.389; 0.034)  *Alistipes* (0.470; 0.009)  *Akkermansia* (0.526; 0.003) | *Parabacteroides* (0.441; 0.028) |
| Valeric | *Faecalibacterium* (-0.417; 0.023)  *Alistipes* (0.408; 0.026)  *Akkermansia* (0.423; 0.021) | *Bacteroides* (0.468; 0.019)  *Parabacteroides* (0.537; 0.006)  *Blautia* (0.586; 0.002)  *Streptococcus* (-0.508; 0.010)  *Roseburia* (0.460; 0.022) |
